# Northern Sea Cucumber (*Cucumaria frondosa*): A Potential Candidate for Functional Food, Nutraceutical, and Pharmaceutical Sector

**DOI:** 10.3390/md18050274

**Published:** 2020-05-22

**Authors:** Abul Hossain, Deepika Dave, Fereidoon Shahidi

**Affiliations:** 1 Department of Biochemistry, Memorial University of Newfoundland, St. John’s, NL A1B 3X9, Canada; abulh@mun.ca; 2 Marine Bioprocessing Facility, Centre of Aquaculture and Seafood Development, Fisheries and Marine Institute, Memorial University of Newfoundland, St. John’s, NL A1C 5R3, Canada

**Keywords:** *Cucumaria frondosa*, geographical distribution, market, bioactive compounds, health benefits

## Abstract

Sea cucumber (*Cucumaria frondosa*) is the most abundant and widely distributed species in the cold waters of North Atlantic Ocean. *C. frondosa* contains a wide range of bioactive compounds, mainly collagen, cerebrosides, glycosaminoglycan, chondroitin sulfate, saponins, phenols, and mucopolysaccharides, which demonstrate unique biological and pharmacological properties. In particular, the body wall of this marine invertebrate is the major edible part and contains most of the active constituents, mainly polysaccharides and collagen, which exhibit numerous biological activities, including anticancer, anti-hypertensive, anti-angiogenic, anti-inflammatory, antidiabetic, anti-coagulation, antimicrobial, antioxidation, and anti- osteoclastogenic properties. In particular, triterpene glycosides (frondoside A and other) are the most researched group of compounds due to their potential anticancer activity. This review summarizes the latest information on *C. frondosa*, mainly geographical distribution, landings specific to Canadian coastlines, processing, commercial products, trade market, bioactive compounds, and potential health benefits in the context of functional foods and nutraceuticals.

## 1. Introduction

Sea cucumber belongs to the class of Holothuroidea and the phylum of Echinodermata; it is globally found in deep seas and benthic areas. Due to multiple biological activities, it has been widely consumed in China, Korea, Japan, Malaysia, Indonesia, and Russia. It has a leathery skin and a soft and cylindrical body containing a single branched gonad. Sea cucumber contains very low fat and cholesterol, but a high protein content [1]. There are around 1500 species of sea cucumber found around the world [2] and about 100 of them are well known for human consumption [3]. The most important commercial species are *Apostichopus japonicus, Acaudina molpadioides, Actinopyga mauritiana, Cucumaria frondosa, Cucumaria japonica, Holothuria forskali, Holothuria polii, Holothuria nobilis, Holothuria tubulosa, Isostichopus badionotus*, and *Pearsonothuria graeffei*. The most common sea cucumbers found in the North Pacific and North Atlantic areas are *Cucumaria frondosa*, *Parastichopus californicus*, *Cucumaria japonica*, and *Parastichopus parvimensis*. In particular, *Cucumaria frondosa* is known as orange-footed sea cucumber, which is the most abundant and broadly distributed species along the east coast of Canada. 

Sea cucumber has received greater attention due to its potential therapeutic benefits and as a marine food product. In addition, it has gained increasing interest as a functional food ingredient due to the availability of its biologically active compounds with medicinal properties. Sea cucumber has an impressive nutritional profile including protein (mainly collagen), lipid (mostly omega-3 and omega-6 fatty acids), vitamins A, B1 (thiamine), B2 (riboflavin), B3 (niacin), and minerals, mainly magnesium, zinc, calcium, and iron [4,5,6,7,8]. Moreover, it contains numerous bioactive compounds, namely saponins [9], glycosaminoglycans [10], chondroitin sulfate [11], sulfated polysaccharides [12,13], fucoidan [14,15], phenolics [16], peptides [17], lectins [18], cerebrosides [19,20], sterols [21], and both the omega-3 and omega-6 fatty acids [22]. As a result, it has been used as a tonic food and folk medicine in Eastern Asia to cure numerous ailments.

East Asian consumers consider sea cucumber as the most luxurious and nutritious food and have used it as a traditional remedy to cure hypertension, rheumatism, asthma, cuts and burns, joint pain, back pain, wound injuries, kidney problem, reproductive disorder, impotence, and constipation [1,23]. The chemical compounds isolated from different sea cucumbers demonstrate unique biological and pharmacological properties such as anticancer [4,24,25], anti-angiogenic [26], anticoagulant [10,27,28], anti-inflammatory [29,30], anti-hypertension [31], antimicrobial [32,33], antithrombotic [28], antioxidant [34,35], antitumor [29], as well as wound healing activities [36]. Particularly, glycosaminoglycan from *C. frondosa* shows heparin-like anticoagulant activity [37]. In addition, sea cucumber-derived bioactive components can be applied to the mouth, face, hands, feet, hair, nails, joints, scalp, and different sensitive body parts as novel cosmetic ingredients [38]. Moreover, dry tablets obtained from the body wall of sea cucumber are broadly used in Asia and the USA for physiological and nutraceutical benefits, particularly for improving sexual performance [5]. In addition, people of Malaysia consume sea cucumber skin extracts to cure hypertension, asthma, wound healing, cuts, and burns [39,40]. Despite the growing interest and demand in sea cucumber, *C. frondosa* has not yet been fully explored compared to other species for potential use as a nutraceutical and functional food ingredient.

To the best of our knowledge, this is the first literature review that attempts to summarize the up-to-date research status about orange-footed sea cucumber (*Cucumaria frondosa*) for its bioactive compounds and their functions. It also discusses the nutritional, biological, and pharmacological properties of *C. frondosa* to highlight its potential use for functional foods as well as nutraceuticals. Moreover, a general view of the growth and distribution as well as landings specific to Canadian coastlines, processing, commercial products, and the trade market of *C. frondosa* is presented. Additionally, an overview of the extraction, isolation, and analysis of biomolecules present in orange-footed sea cucumber is provided. 

## 2. Description, Growth, and Distribution 

**Figure 1:**
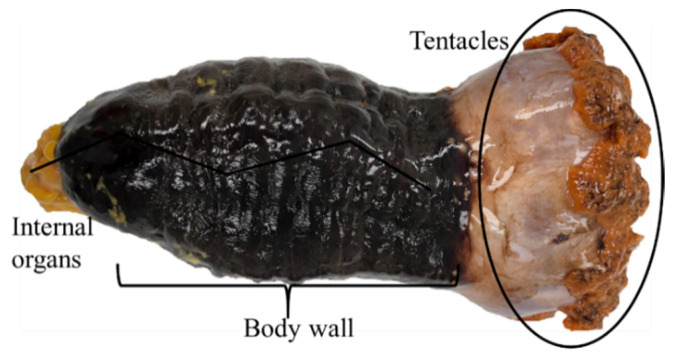
Body parts of *Cucumaria frondosa*.

The orange-footed sea cucumber (*C. frondosa*) is widely distributed in the north Atlantic, mainly in the nearshore parts, and in the Barents Sea along the coast of the Russian Federation. They are soft-bodied, cucumber-like, with leathery skin, elongated, and worm-like body. The mouth is surrounded by aquapharyngeal bulb/tentacles/flower at one end of the body and an anus at the opposite end (Figure 1). It can grow to a maximum length of 40–50 cm, a width of 10–15 cm, and a weight of 100 to 500 g. The body wall is the main part (up to 50% of the total body weight) of this species, which contains around 85% moisture. Generally, sea cucumbers eat mud or dead particle remains; however, *C. frondosa* feed on phytoplankton, zooplankton, and organic matter by spreading out their tentacles [41,42].

Interestingly, this species can regenerate or renew themselves very quickly, and they may have the ability to restore their lost organs [43]. Sea cucumbers contain collagen (echinoderms collagen) in their skin, so they are able to change their mechanical state (liquid/jelly to solid form) very quickly [44] and, hence, use it as a possible defense mechanism. Moreover, it is assumed that they control their movement by thousands of tiny tube feet and communicate with each other by transferring hormone signals through the water. Due to the plasticity of their physical characteristics, morphometrics, including length, weight, and age are quite challenging to determine [45]. Generally, *C. frondosa* (orange footed sea cucumber), pumpkins or phenix sea cucumber are harvested from May to November in Atlantic Canada. Moreover, the growth rate of *C. frondosa* is slower compared to other sea cucumber species with an average growth rate of 2 mm per month. Furthermore, the growth rate is dependent on temperature, light, salinity, depth, and level of disturbance [46]. However, due to the small size and thin body wall, *C. frondosa* is still considered a low-grade product compared to other commercial species of sea cucumber [47]. 

**Figure 2:**
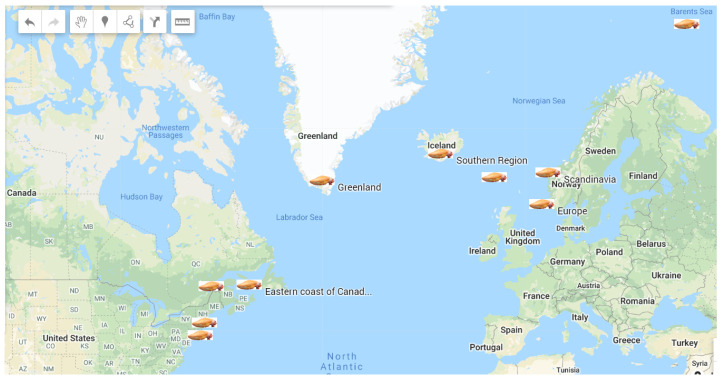
Distribution of the Northern sea cucumber (*Cucumaria frondosa*).

The distribution of *C. frondosa* ranges from the eastern coast of Canada (Gulf from the lower tide limit to the deepest area and St. Lawrence Estuary), southwest coast of New England, down to the coast of northern Europe, southern Iceland, and the coast of Greenland, Scandinavia, and the Faroe Islands (Figure 2). Particularly, it is found from lower inter-tidal and cold tide-pools to sub-tidal down to 1000 ft and from the Arctic to Cape Cod. These sea cucumbers are most abundant at strong currents and depths region (30 to 300 m), and they prefer to live in rocky (corals and seaweeds) or in mixed substrates (stone, sand, gravel, and shells) [48,49,50].

## 3. Canadian Landings, Processing, Commercial Products, and Trade Market

**Figure 3:**
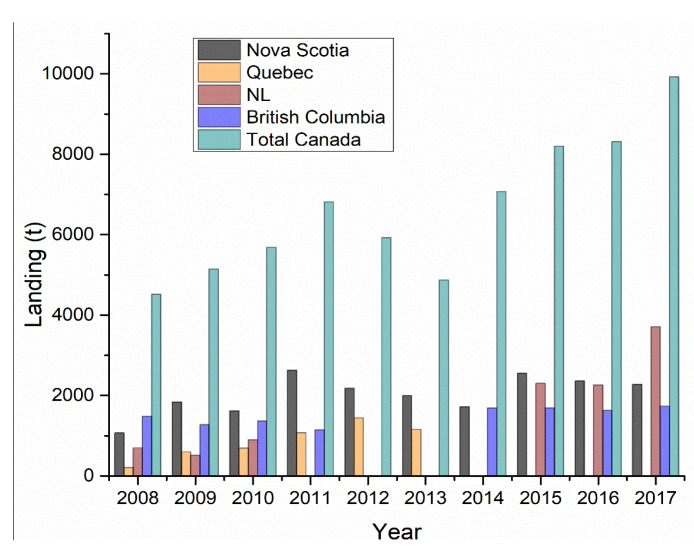
Volume of Atlantic and Pacific Coasts commercial landings (metric tons, live weight).

**Figure 4:**
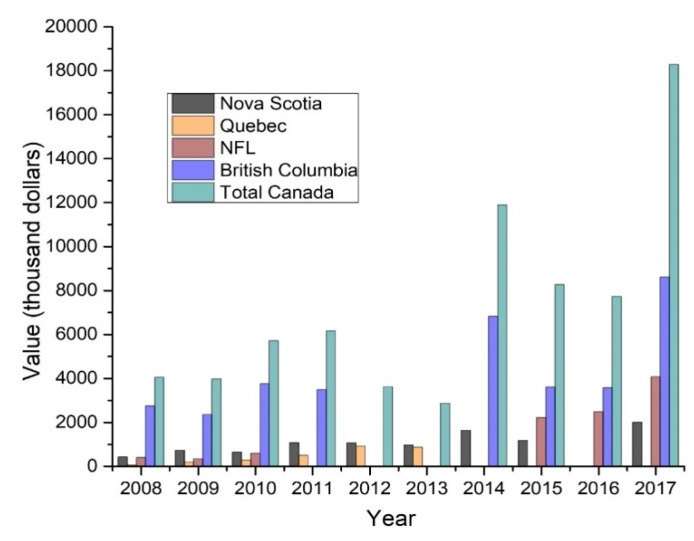
Value of Atlantic and Pacific Coasts commercial landings (thousand dollars, C$).

According to the Department of Fisheries and Oceans (DFO) of Canada [51], the volume of Atlantic and Pacific Coast commercial landings of sea cucumber has doubled over the last decade (Figure 3). In particular, the volume was increased over five times in Newfoundland and Labrador from 2008 to 2017 (698 to 3707 metric tons). Moreover, from 2008 to 2017, the Canadian landing increased from 4516 to 9922 metric tons with a value of $18.3 million (Figure 4). *C. frondosa* inhabits up to a depth of 100 m in waters off Newfoundland and Labrador (NL). In 2003, a sea cucumber drag fishery was established on the St. Pierre Bank, under the New Emerging Fisheries Policy. Initially, it was allocated of 454 tons, then it was increased to 612 tons in 2005, 907 tons in 2010, and the allocation was increased to 2242 t in 2013 [42]. In 2017, Newfoundland and Labrador contributed 37.36% (3707 metric tons) of the total sea cucumber landings in Canada and accounted for $4 million in revenue.

The storage and transport of sea cucumber after harvesting are challenging tasks due to the ability to autolyze upon removal from seawater or under stress. Autolysis is a physiological process where body wall/dermis degrade through protein breakdown (endogenous proteases) which may lead to a change in the texture and organoleptic properties of the meat [52]. Generally, sea cucumber is placed in seawater/ice/salt immediately after harvesting. Gianasia, Hamel, and Mercier [52] reported that iced seawater provided the best storage condition, whereas common traditional methods, including icing and salting, yielded the highest rates of mortality and skin necrosis. Moreover, digestive tract secretes various enzymes, mainly chymotrypsin, trypsin, and, cathepsin, after harvesting, which act in the hydrolysis of one of the main components of the body wall (collagen) [53], thus, leading to the deterioration of the body wall and loss of final products’ quality. The development of new components, following protein breakdown, lipid oxidation, and enzyme secretion, change the color, flavor, odor, and texture of the sea cucumber and result in economic loss [52]. 

**Figure 5:**
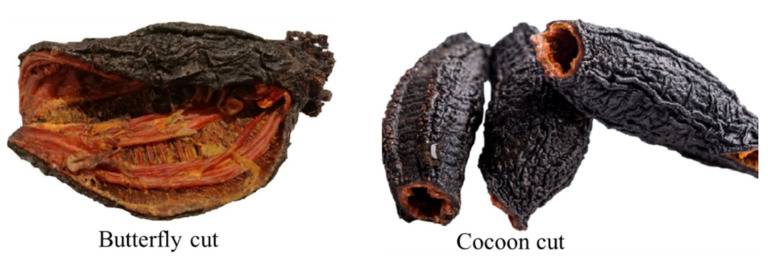
Butterfly cut (dried with meat) and cocoon cut (dried with meat).

Sea cucumber processing steps consist of eviscerating and gutting, followed by cleaning and freezing/cooking/drying. The body wall is the primary product of sea cucumber; sometimes the longitudinal muscles (referred to as meat) are also separated from the body wall. Some industries separate tentacles (flower) alone and sell as a dried form; however, internal organs are considered as a processing waste. The most common Atlantic sea cucumber products are butterfly cut (skin with/ without meat, dry or frozen), cocoon cut (skin with meat, dry or frozen), sliced skin (frozen/ dried), and raw meat (frozen) (Figure 5). Sometimes, Individually Quick Frozen (IQF) is used to store the whole sea cucumber after blanching. The butterfly cut process consists of cutting the cucumber along its body wall to remove internal organs (gutting), the resultant sea cucumber is “unrolled”, which provides a butterfly-like shape. On the other hand, the cocoon cut process consists of cutting the flower off to create an opening for gutting. Gutting can be done through a uniquely designed cleaning tool or using a vacuum. Cocoon cut provides a higher value in the world market over the butterfly cut (longitudinal cut) due to its elongated and undamaged tubular shape (personal communication and visit to local sea cucumber industries).

**Figure 6:**
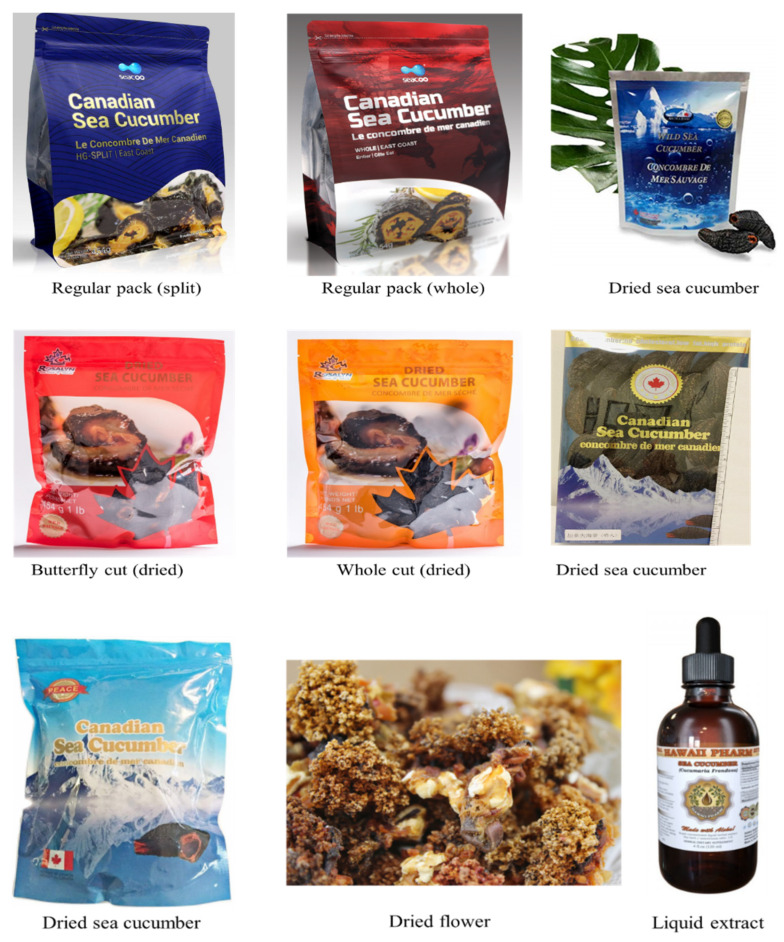
Most common *C. frondosa* products in the market.

*C. frondosa* is exported in the world market as a frozen, cooked-salted, cooked-dried, and cooked-salted-dried form. However, most of the global trade market is in the dried product form while a small quantity sells as frozen and fresh products. Around 90% of this trade takes place in China, Hong Kong, Japan, Korea, and Singapore. Wild sea cucumbers, which are harvested from cold and Arctic waters, have a greater value in the Asian market over those from aquaculture, mainly tropical and warm waters. Depending on the thickness of the body wall, texture, skin color, and processing integrity, the value of dried sea cucumber may vary widely, and the price per kilogram can range from $20 to $360 US. Table 1 and Figure 6 show the commonly found products derived from sea cucumber (*Cucumaria frondosa*) in the marketplace.

## 4. Proximate Composition

Very few studies have so far been performed on the proximate composition of *C. frondosa*. From a nutritional point of view, *C. frondosa* is an ideal tonic food and has an impressive nutritional profile such as vitamins, minerals, carbohydrates, amino acids, and fatty acids. Zhong, Khan, and Shahidi [54] reported that the content of moisture, protein, lipid, ash, and carbohydrate in fresh whole sea cucumber were approximately 90.5, 5.5, 0.8, 3.5, and 1.5%, respectively. Both essential and non-essential amino acids (17 of them) were present along with a high amount of free amino acids. In particular, fresh whole sea cucumber contains glutamic acid (57.5 mg/g), lysine (30.6 mg/g), leucine (28.3 mg/g), glycine (29.8 mg/g), asparagine (27.8 mg/g), along with a considerable amount of alanine, arginine, proline, and valine. However, fresh whole sea cucumber had lower amino acids and fatty acids contents than those with internal organs removed (Table 2). Moreover, eicosapentaenoic acid (EPA) was the predominant fatty acid in *C. frondosa* compared to docosahexaenoic acid (DHA). On the other hand, Mamelona, Saint-Louis, and Pelletier [55] stated that Atlantic sea cucumber viscera contained approximately 92.3% moisture, 4.5% protein, 2% fat, 0.7% ash, and 0.3% carbohydrate. Most of the essential and non-essential amino acids were also present with a high amount of glutamic acid, aspartic acid, and arginine. Moreover, Atlantic sea cucumber viscera are a rich source of polyunsaturated fatty acids (PUFA, about 44%) with 24% saturated fatty acids (SFA) and 30% monounsaturated fatty acids (MUFA). The gonad and muscle tissues of *C. frondosa* had a significantly higher amount of lipid and fatty acids (EPA and DHA) compared to other body parts [50]. They also reported that a considerable amount of lipids (3.40 ± 0.28 mg/g w/w); mainly DHA, palmitic acid, and EPA was present when *C. frondosa* fed on a fish eggs diet. However, viscera contained a high level of essential (Cu, Fe, Zn, K, Na, Mn, As, Mg, Se, Ni, and Ca) and a very small amount non-essential (Cd, Co, and Pb) trace elements, and vitamins (niacin, pantothenic acid, alpha-tocopherol, riboflavin, thiamine, and folates). Theses minerals stimulate the metabolism of the body, promote healthy growth, and assist in lowering the blood sugar level [55]. Therefore, *C. frondosa* is considered as a rich source of nutrients, including vitamins, and minerals, in the marine food industry. 

## 5. Bioactive Compounds and Methods of their Extraction and Isolation 

**Figure 7:**
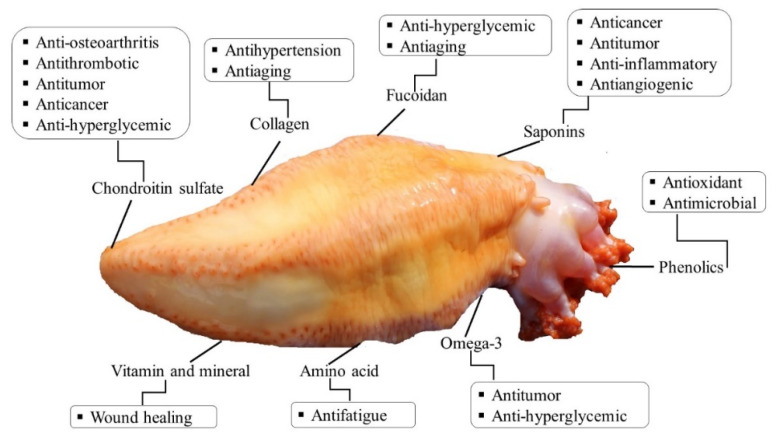
Bioactive compounds of *C. frondosa* and their potential health benefit.

Orange-footed sea cucumber is one of the potential marine sources with value-added compounds that could have medicinal properties. The most common bioactive compounds found in *C. frondosa* are triterpene glycosides, polysaccharides (fucosylated chondroitin sulfate), cerebrosides, saponins, carotenoids, collagens, phenols, PUFA, and other bioactive compounds [5,56,57]. The major bioactive compounds of Atlantic sea cucumber are shown in Figure 7 and explained in the following subsections. This figure does not indicate the location of bioactives, which is shown in the Section 6.

### 5.1. Polysaccharides

**Figure 8:**
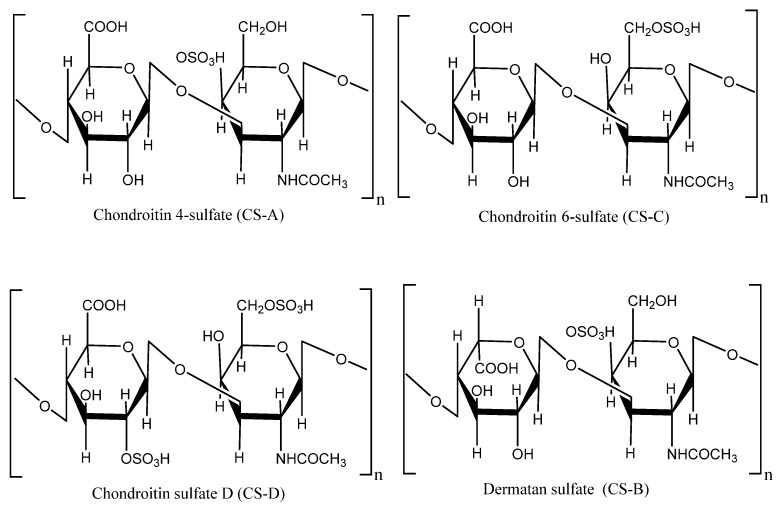
Structure of repeating disaccharide units in chondroitin sulfate.

Over the past few decades, there has been considerable research on marine creatures, and many researchers have focused their attention on polysaccharides derived from marine organisms. Polysaccharides from marine organisms possess potential health benefits, hence are used in food, nutraceuticals, pharmaceuticals, and cosmetic industries. Sea cucumber has ultimately become one of the primary sources of polysaccharides due to its wide range of pharmacological and biological activities. The body walls of *C. frondosa* contain a high amount of acidic polysaccharides, particularly sulfated polysaccharides (fucosylated chondroitin sulfate) [56,57]. Interestingly, the structure of sulfated polysaccharides identified from sea cucumber is different from other vertebrates, invertebrates, and algae [12]. There are two types of polysaccharides identified in *C. frondosa*: (a) fucosylated chondroitin sulfate and (b) fucan [57,58]. Fucosylated chondroitin sulfate (FCS) is a unique glycosaminoglycan found in sea cucumber, and its bioactivity depends on the sulfation pattern of monosaccharide composition. Glycosaminoglycans (GAGs) are sulfated, linear, viscous, lubricating, and negatively charged polysaccharides, which are found in mammalian as well as avian species. Bioactivity of FCS depends on the position of sulfate, the degree of sulfation, and the distribution of branches along the backbone. However, chondroitin sulfate (CS) consists of repeating disaccharide unit of glucuronic acid and *N*- acetylated galactosamine, which is attached by tetrasaccharide linkage with protein cores. CS presents as chondroitin sulfate A (CS-A) which is sulfated at *O*-4 of *N*-acetylgalactosamine (GalNAc), CS-C at *O*-6 position of GalNAc, CS-D at 6 position of GalNAc and glucuronic acid (GlcA), CS-E at 4 and 6 of GalNAc, and CSB which is known as dermatan sulfate (DS) (Figure 8) [59]. Ustyuzhanina et al. [57] isolated fucosylated chondroitin sulfate from the body wall of *C. frondosa* and found that it comprised of chondroitin sulfate A and E together with the disaccharide repeating units →3)-ẞ-D-GalNAc4S6S-(1→4)-ẞ-D-GlcA3S-(1→ and →3)- ẞ-D-GalNAc4S-(1 → 4)-ẞ-D-GlcA3S-(1→. They also detected three types of branches in *C. frondosa*; two of them were α-L-Fucp3S4S and α-L-Fucp2S4S link to *O*-3 of GlcpA residues, whereas the last one was per-*O*-sulfated α-L-Fucp attached to *O*-6 of GalpNAc residue. On the other hand, Kale et al. [56] stated that the monosaccharides composition of *C. frondosa* as *N*-acetylneuraminic acid, *N*-acetylgalactosamine, *N*-acetylglucosamine, glucuronic acid, mannose, fucose, glucose, and galactose.

The method used to extract the fucosylated chondroitin sulfate (FCS) from sea cucumber includes many steps, such as chemical hydrolysis, proteolytic digestion to release CS, DS, and other GAGs, elimination of proteins and recovery of CS, fractionation of CS, and purification of CS. Chemical hydrolysis and proteolytic digestion can be achieved by using NaOH, cysteine or guanidine HCl, non-ionic detergents, quaternary ammonium salts (cetylpyridinium chloride), urea, potassium thiocyanate or alcoholic solution, whereas recovery of CS can be obtained using trichloroacetic acid. The purification of CS is conducted by using ion exchange, gel filtration, and size exclusion chromatography. The extraction process can be summarized by dilute alkali-enzymatic hydrolysis (alkali solution and papain, trypsin, Alcalase, subtilisin or pepsin), mechanochemically-assisted extraction method, ultrasound-assisted extraction method, organic solvent precipitation, fractionation by precipitation with quaternary ammonium salts and column chromatography [60]. However, many alternative extraction methods have recently been established such as application of high hydrostatic pressure [61], combination of hydrogen peroxide and copper ions [62], tissue autolysis [63], and ^60^Co irradiation [64], among others. 

### 5.2. Fucoidan

Fucoidan is one of the most important bioactive components of the sea cucumber body walls. This polysaccharide is comprised of L-fucose and sulfate groups. Chain conformation of polysaccharides substantively affects their bioactivities and physicochemical properties [65]. More than 20 species of algae fucoidan have been examined and used in the functional food industries [66]. However, sea cucumber fucoidan has been reported to have antithrombotic and anticoagulant properties [67], inhibition of osteoclastogenesis [68], and protection from gastric damage [69]. Moreover, Wang et al. [70] reported that fucoidan from *C. frondosa* exhibits anti-hyperglycemic properties, which significantly decreases fasting blood glucose and insulin levels, and increases insulin and glucose tolerance in insulin-resistant mice. Moreover, it has been suggested that *C. frondosa* fucoidan could be used as a complementary treatment for diet-induced type 2 diabetes. On the other hand, Hu et al. [58] explained that *C. frondosa* fucoidan significantly prevented high-fat high-sucrose diet injured pancreatic islets, decreased insulin, tumor necrosis factor (TNF)–α, and blood glucose levels, and enhanced adiponectin level. In addition, they proposed that it prevents pancreatic islets apoptosis through inhibition of the mitochondrial pathway. The major extraction method is enzymatic hydrolysis, mainly using papain, followed by cetylpyridinium chloride precipitation [58,70]. The Sepharose Q Fast Flow column is used to purify the crude sulfated polysaccharide.

### 5.3. Collagen

Collagen, an abundant protein in animals, is mainly spread in the extracellular matrix, inner dermis, tendon, bone, cartilage, ligament, and other connective tissues, which supports an extracellular framework for strength and flexibility [71]. Moreover, 30% of the body protein content is collagen; animal-derived collagen is broadly used in food, pharmaceuticals, and cosmetics industries. Typically, collagen fibers are hardly soluble, and the most common form of collagen is type I (fibrillar collagen) [72]. However, gelatin is a soluble form of collagen, which is obtained by partial hydrolysis of collagen [71]. Due to gel-forming and water-binding properties, gelatin is widely used in food, pharmaceutical, cosmetic, and photography industries as emulsifiers, colloid stabilizers, foaming agents, microencapsulating, and biodegradable film-forming material. Nowadays, the most common raw material for extracting collagen is pigskin (46%), bovine hide (29.4%), pork and cattle bones (23.1%), and aquatic animals at 1.5% [71]. Due to food safety and religious restriction, extraction of collagen from porcine and other mammalian sources is still limited. The potential foot-and-mouth disease of bovine and outbreaks of porcine spongiform encephalopathy have provoked some anxiety among health-conscious consumers [73]. Besides, collagen/gelatin obtained from cows that are not religiously slaughtered and pigs are not acceptable to Muslims and Jews. Collagen from Beef is also prohibited for Hindus [74]. Therefore, marine sources have become a new trend for the extraction of collagen due to being free from such limitations. However, few studies have been researched on extraction of collagen from chicken cartilage, rat tail tendon, kangaroo tail, equine tendon, duck feet, alligators bone and skin, sheepskin, bird feet, frog skin, and some marine sources [75,76]. 

**Figure 9:**
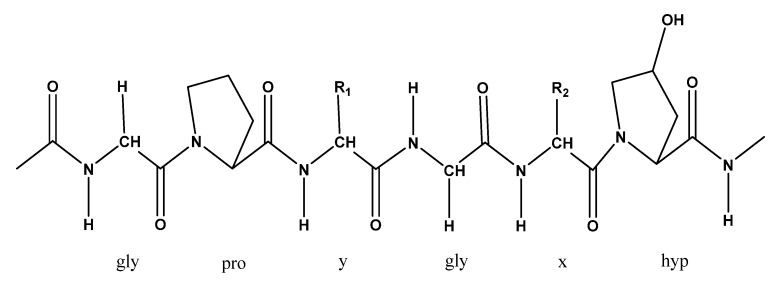
Primary amino acid sequence of type I collagen.

The most valuable edible part of sea cucumber is the body wall, which represents around 50% of the body weight, mainly considered to consist of collagen and mucopolysaccharides. Collagen is reported to be the major protein of sea cucumber with approximately 70% of insoluble collagen fibrils present in the body wall, while the crude protein in dried sea cucumber estimated around 83% of its dry weight [23,77]. The most abundant type of collagen in sea cucumber is collagen fibrils of echinoderms and type I collagen, which is symmetrically spindle-shaped and short in length [7,78]. *C. frondosa* has been reported to serve as a good source of thermally stable collagen due to the presence of type I collagen (Figure 9) [79,80]. Moreover, the principal collagen of *C. frondosa* dermis forms α1 trimers that are covalently linked with the main GAGs found in the dermis. In contrast, it was found that the body wall of *C. frondosa* contains less than a fraction of one percent collagen (our unpublished work). This could be related to the unique feeding habits (mainly phytoplankton, zooplankton, and organic matters) of this species compared to other species (mostly mud or dead particles). Generally, both conventional and novel methods are used for the extraction of collagen from sea cucumber. The conventional methods include chemical hydrolysis (acid and alkali hydrolysis) and enzymatic hydrolysis (trypsin, chymotrypsin, pepsin, papain, bromelain, ficin, proteinase K, collagenase, Neutrase, Alcalase, Protamex or Flavourzyme), whereas novel methods include ultrasound-assisted and pressurized liquid extraction procedures. These newly emerging and novel techniques are considered to offer the best way compared to the conventional methods, due to being safe, economical, time saving, and environmentally-friendly approaches. Lastly, collagen may be purified by using different chromatographic techniques, such as size exclusion chromatography, high-performance liquid chromatography (HPLC), and ion-exchange chromatography.

### 5.4. Saponins

Saponins are triterpene glycosides and secondary metabolites created by holothurians. They are broadly distributed in plants, animals, and marine organisms (holothurians and sponges) [81]. Saponins play a crucial role in chemical defense as well as pharmacological activities. Approximately 300 triterpene glycosides have been identified and categorized from many species of sea cucumbers, which are named as holostane and nonholostane. Saponins (triterpene glycosides) comprise a carbohydrate chain of up to six monosaccharides, including D-xylose, D-glucose, 3-*O*-methyl-D-xylose, 3-*O*-methyl-D-glucose, and D-quinovose. Moreover, about 60% of the triterpene glycosides identified from sea cucumbers have sulfate groups attached to the monosaccharide groups of the carbohydrate chain [82]. 

**Figure 10:**
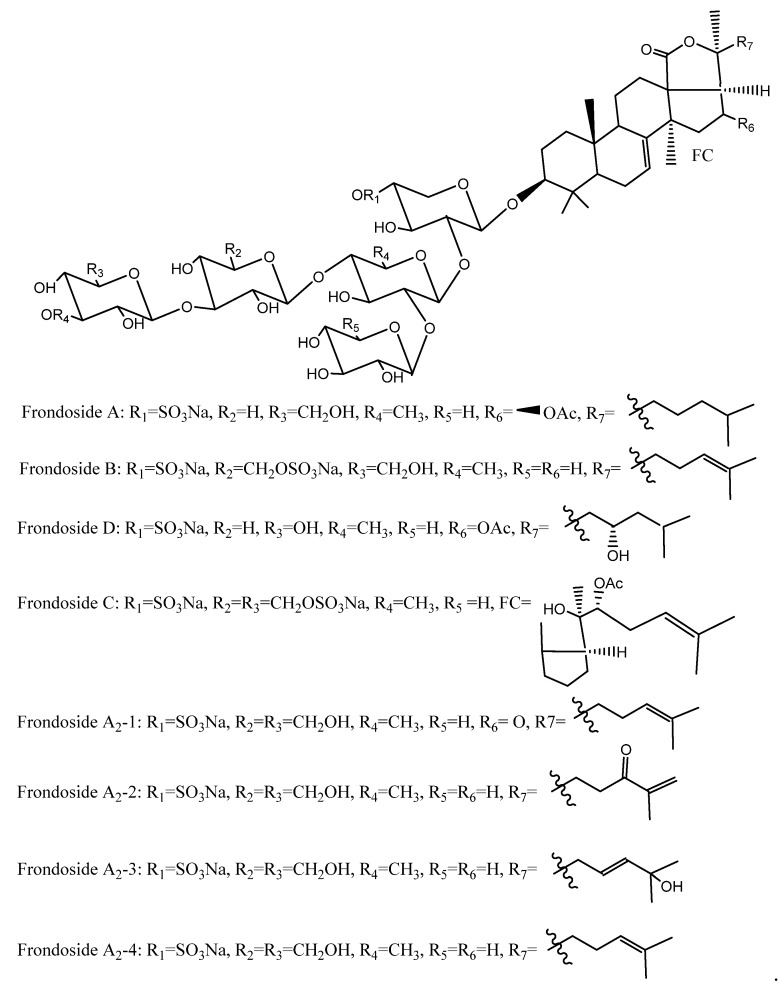
Chemical structures of different frondosides [82].

The saponin isolated from sea cucumbers, typically known as holothurin, is well known as frondoside A. The *C. frondosa* species contains various types of triterpene glycosides, mainly frondoside A, frondoside B, frondoside C, isofrondoside C, frondoside A_2_-1, frondoside A_2_-2, frondoside A_2_-3, frondoside A_2_-4, frondoside A_2_-6, frondoside A_2_-7, frondoside A_2_-8, frondoside A_7_-1, frondoside A_7_-2, frondoside A_7_-3, and frondoside A_7_-4 (Figure 10) [82,83,84,85,86,87,88,89]. Moreover, *C. frondosa* comprises a very complex mixture of monosulfated frondoside A, disulfated frondoside B, and trisulfated frondoside C [83,84,85,86,87,88]. Furthermore, Findlay et al. [85] reported that the major saponin in *C. frondosa* is frondoside A. They also found three novel oligosaccharides, namely frondoside B, frondoside D, and dimeric pentasaccharide frondecaside. Yayli [86] categorized three other minor saponins from *C. frondosa*, namely frondoside F, frondoside E_1_, and frondoside E_2_, though Kalinin et al. [87] stated that the structure of these glycosides and frondecaside is uncertain. These compounds exhibited various biological properties, including cytostatic, hemolytic, antiviral, antiprotozoal, antifungal, anticancer, antineoplastic, and antitumor activities [26,90]. Saponins have been purified by various techniques including liquid-liquid extraction with multiple solvents, high-performance liquid chromatography (HPLC), solid-phase extraction, or chromatography (resins or silica gel). Finally, ^1^H NMR and ^13^C NMR spectra are used to identify the structure of the oligosaccharide moiety [86,88].

### 5.5. Phenolic Compounds

Phenolic compounds are powerful antioxidants that are broadly distributed in plants as well as seaweeds, and marine invertebrates. Their effects as beneficial antioxidants to shield the human body from many chronic diseases has been of particular interest. These compounds are partially responsible for flavor, color, bitterness, astringency, and nutritional value of foods [91,92]. Generally, plant-based foods contain around 60 times more antioxidants than their animal-based counterparts. However, sea cucumber, particularly *C. frondosa* contain a significant amount of phenolics with moderate antioxidant activity even though it is an animal species [16,54,93,94,95]. Due to the absorption of phenolics from phytoplankton, marine invertebrates may possibly serve as a rich source of phenolics including flavonoids, anthocyanidins, anthocyanins, and tannins [54].

It has been reported that the different body parts (muscles, gonads, digestive tract, and respiratory apparatus) of *C. frondosa* contain a significant amount of phenolics (22.5 to 236.0 mg gallic acid equivalents (GAE)/100 g dw) and flavonoids (2.9 to 59.8 mg of rutin equivalents/ 100 g dw) with oxygen radical absorbance capacity (ORAC) values 140 to 800 µmol of Trolox equivalents/g dw [16]. Moreover, the same study stated that the highest level of phenolics was obtained from the digestive tract using acetonitrile-rich fractions and ethyl acetate extracts, whereas the highest amount of flavonoids was found in the gonads when considering water-rich and acetonitrile-rich fractions. Similarly, Zhong et al. [54] reported that *C. frondosa* show the highest ORAC (2.60 ± 0.04 mmol of Trolox equivalents/g dw) and 2,2-diphenyl-1-picrylhydrazyl (DPPH) (7.48 ± 0.10 µmol of Trolox equivalents/g dw) activity in rehydrated sea cucumber (mainly internal organs) compared to fresh counterparts, whereas the fresh *C. frondosa*, with or without internal organs, contained a significant amount of phenolics (1.08 mg GAE/g dw) compared to the rehydrated samples. In another study, Mamelona and Pelletier [96] described that the viscera of *C. frondosa* exhibited the highest antioxidant activity in ORAC assay in ethanol extracts compared to isopropanol, methanol, and water extracts at 60 °C of extraction by pressure liquid extraction (PLE) method. Additionally, the same study demonstrated that PLE allowed better extraction of α-tocopherol (220 µg/g), total carotenoids (60 mg/g), and total phenols (894 µg/g) by ethanol followed by isopropanol, methanol, and water at 60 °C. In addition, free, esterified, and insoluble-bound phenolics were extracted from different body parts of *C. frondosa*, and their antioxidant activity was determined. Results suggested that the free fraction was the most predominant form of phenolics in all the selected body parts. Moreover, the highest amount of phenolics and antioxidant property was detected in tentacles (flower), followed by internal organs and body wall [97].

It has been reported that the major phenolic compound in sea cucumbers (*Holothuria atra* and *Holothuria arenicola*) is chlorogenic acid (up to 93 wt%), but other phenolics present were pyrogallol, coumaric acid, rutin, and catechin [98]. However, to the best of our knowledge, no other published reports are available on the phenolics profile in *C. frondosa*. 

## 6. Potential Biological Activities and Medicinal Effects

Sea cucumber is recognized as a folk medicine and a traditional food globally, particularly in East Asia. However, its specific constituents and their biological functions are yet to be examined. So far, its anti-angiogenic, antithrombotic, anticoagulant, anticancer, antitumor, anti-inflammatory, antihypertension, antifungal, antimicrobial, and antioxidant properties have been investigated. Additionally, it has been used for the treatment of asthma, stomach ulcer, rheumatism, kidney diseases, wound healing, nourishing the body, and as moisturizing agent. Biological and medicinal benefits of *C. frondosa* are summarized in Table 3. 

### 6.1. Anticancer Activities

Approximately 60% of permitted cancer treatment drugs are isolated from natural origin such as plant, animal, and marine sources [105]. On the average, 14,000 biologically active compounds have been identified from marine resources, recommending it is a rich sector for isolating novel pharmacologically active substances for discovering new anticancer drugs [106]. In 1952, Nigrelli [107] described the anticancer activities of Bahamian sea cucumber (*Actinopyga agassizi*) glycoside for the first time. Later, many sea cucumber species have been studied for the anticancer mechanism in more in-depth, predominantly *C. frondosa* (frondoside A) (Table 4).

Li et al. [108] described that the novel terpenoid (frondoside A) of *C. frondosa* displays a growth inhibitory characteristic against pancreatic cancer cells by prompting apoptosis via cascade activation and mitochondrial pathways. Moreover, Liu et al. [25] studied the anticancer activity of low-molecular-weight fucosylated chondroitin sulfate (LFCS) from *C. frondosa* and demonstrated that LFCS significantly inhibited Lewis lung carcinoma growth and metastasis in a dose dependent manner. Besides, LFCS remarkably inhibited the promptness of ERK1/2/p38 MAPK/NF-kB pathway, which played a key role in the expression of matrix metalloproteinases. In addition, the anticancer mechanism was associated with p53/p21-induced cell cycle arrest, vascular endothelial growth factor mediated angiogenesis, caspase-3-induced apoptosis, and tissue inhibitor of metalloproteinase/ matrix metalloproteinase-mediated metastasis by the ERK1/2/p38 MAPK/NF-kB pathway. However, Al Shemaili et al. [101] investigated the effects of frondoside A with gemcitabine on pancreatic cancer (AsPC-1 and S2013) and suggested that a combination of frondoside A (100lg/ kg/day) and gemcitabine (4 mg/kg/dose) was more effective compared to the single drug alone. In another study, Al Shemaili et al. [109] compared the frondoside A with the frondoside B and C, and the aglycone compound on growth inhibitory effects; results suggested that frondoside A was more effective anticancer agent than other frondosides. Additionally, frondoside A significantly suppressed the growth of pancreatic cancer cells (EC_50_ of ~1 μM), while frondoside B was less effective (EC_50_ ~2.5 μM), and frondoside C and the aglycone had no activity. Besides, frondoside A at 100 μg/kg/day noticeably suppressed the growth of cancer xenografts in mice.

Another study described the isolation of frondanol A5 from *C. frondosa* extracts and suggested it as a possible chemopreventive agent against colon cancer [110]. They used aberrant colonic crypt foci as an efficacy marker to measure the proliferation of expression, and frondanol A5 (10–120 μg/mL) in the HCT-116 cell line was also used for its apoptotic and growth-inhibitory effects. In this, dietary administration of 150 and 450 ppm of frondanol A5 decreased the total colonic aberrant crypt foci formation, which is prompted by azoxymethane (around 34 to 55%) and multicrypt aberrant foci (about 48 to 68.5%) in a dose-dependent manner. Frondanol A5 comprises various anticancer substances, such as monosulfated triterpenoid glycoside frondoside A, EPA, disulfated glycoside frondoside B, 12-methyltetradecanoic acid, trisulfated glycoside frondoside C, and fucosylated chondroitin sulfate. On the other hand, Roginsky et al. [111] studied the activity of frondanol-A5P, a polar fraction from *C. frondosa* on anticancer effects in S2013 as well as AsPC-1 human pancreatic cancer cells. As a result, frondanol-A5P inhibited proliferation and initiated cell cycle arrest at G_2_/M phase in both cell lines with the declined expression of cyclin A, cyclin B, and cdc25c. In addition, frondanol-A5P prominently improved annexin V binding and initiated caspase-3.

Frondoside A has been researched for breast cancer treatment and considered a potential source for inhibiting breast cancer. Al Marzouqi et al. [112] examined the effect of frondoside A on human breast cancer cell survival, tumor growth in nude mice, and migration and invasion in vitro using the human estrogen receptors (ER)-negative MDA-MB-231 cells breast cancer cells. Results suggested that frondoside A improved sub-G1 (apoptotic) cell fractions through increases in p53 followed by the induction of the caspase 3/7 and 9 cell death pathways in breast cancer cells. Additionally, frondoside A (100 μg/kg/day intraperitoneal for 24 days) powerfully declined the growth of tumor xenografts in athymic mice without any toxic effects. Hence, frondoside A may increase the inhibiting of breast cancer cells prompted by the chemotherapeutic agent paclitaxel. On the other hand, frondoside A has possible antimetastatic properties on the syngenic murine model of metastatic breast cancer with cell line 66.1. Moreover, 3H-PGE2 binding assays showed that frondoside A provoked PGE2 binding to both receptors (EP2 and EP4), whereas EP4 receptor showed a much higher affinity. Besides, frondoside A (0.1 and 1.0 μM) suppressed the migration of tumor cells in response to EP2 or EP4 receptors [113]. In another study, Park et al. [114] studied the anti-invasive property of frondoside A against human breast cancer cells and suggested frondoside A as an anticancer agent for metastatic breast cancer. It was found that the frondoside A notably declined TPA-induced colony formation and invasion as well as migration in MBA-MB-231 human breast cancer cells.

It has been reported that frondoside A may inhibit lung cancer including LNM35, NCI-H460-Luc2 LNM35, NCI-H460-Luc2, and A549, as well as breast cancer, including MDA-MB-435, HepG2, and MCF-7 cell proliferation when injected at 0.01–5 µM. Furthermore, when frondoside A was injected at 0.01 and 1 mg/kg into athymic mice with LNM35 lung cancer cells reduce tumor volumes 41 and 43% after 25 days of treatment, respectively. Besides, frondoside A induced inhibition of cell migration, angiogenesis, as well as invasion in vitro [115]. On the other hand, Jin et al. [116] compared the effects of frondoside A with cucumarioside A_2_-2 (from *Cucumaria japonica*) on cell death-inducing capability and reported that both frondoside A and cucumarioside A_2_-2 showed anti-leukemic activity by inducing apoptosis. However, the apoptosis induced by frondoside A was more pronounced and rapid than the cucumarioside A_2_-2-induced apoptosis. 

### 6.2. Antitumor Activities

Antitumor compounds play a vital role against different phases of tumor development, metastasis, and progression. The U.S. National Cancer Institute made a 15-year survey and reported that 4% of marine creatures contained antitumor constituents [24]. Sea cucumbers such as *C. frondosa* contain various antitumor components and show significant inhibitory effects on tumor growth [112]. It has been reported that the tumor volume was reduced by 87% in the mouse model using MDA-MB-231 breast cancer cells when frondoside A was employed [113]. According to Janakiram et al. [118], frondanol A5 suppresses intestinal tumors through a rise in innate immune responses against tumors and apoptosis. Additionally, the macrophages displayed an increment GILT expression in the treatment sets, resulting in a modulation of inflammatory cytokines in the tumors. This is because frondanol A5 is responsible for improving immune responses as well as inhibiting intestinal tumors in APC^Min/+^ mice. In another study, Liu et al. [25] isolated low-molecular-weight fucosylated chondroitin sulfate from the body wall of *C. frondosa* and proposed that it reduced the tumor volume and weight significantly in a dose-dependent manner in vivo. Besides, the inhibition rates (1, 5, and 20 mg/kg) of LFCS were 25.8, 33.1, and 47.2%, respectively. Avilov et al. [83] also studied frondoside C, a new triterpene glycoside from *C. frondosa*, which was used to determine the antitumor activity in the cell lines P-388, A-549, HT-29, and Mel-28. In another study, Wang et al. [119] isolated 13 triterpene glycosides from *C. frondosa* as well as *H. scabra*, and evaluated their cytotoxic activities. Results demonstrated that the number of glycosyl residues in sugar chains as well as a side chain in aglycone may influence their cytotoxicity to tumor cells and render selective cytotoxicity. According to another research, phospholipids were extracted from *C. frondosa*, and liposomes were prepared [120]. Results showed that liposomes had antitumor effects in vitro and demonstrated high transport as well as uptake effects in small intestinal epithelial cell models. In addition, liposomes extended the lifespan of S180 ascitic tumor-bearing mice via reducing ascitic fluid volume as well as killing ascitic tumor cells.

### 6.3. Antithrombotic and Anticoagulant Activities

Antithrombotic agents reduce the formation of thrombus, whereas anticoagulants reduce the ability of the blood to clot. The presence of fucosylated chondroitin sulfate (FCS) in the body wall of sea cucumber is associated with the anticoagulant as well as antithrombotic activities. It has been reported that anticoagulant property generally depends on the sulfate content, position, and molecular size of the polysaccharides, chain conformation, and monosaccharide composition [99,121]. However, Liu et al. [99] isolated FCS from *C. frondosa* and Cu^2+^ catalytic free-radical depolymerization to prepare low-molecular-weight fragments. The structure of disaccharide components was analyzed by nuclear magnetic resonance (NMR), and antithrombotic as well as anticoagulant properties were identified with different molecular weights and sulfation patterns. The results demonstrated that low-molecular-weight fragments of FCS exhibited better anticoagulant and antithrombotic properties compared to the native FCS in a rat model. Moreover, molecular weight and degree of sulfation had a more pronounced effect on anticoagulation and antithrombosis than the sulfation pattern.

### 6.4. Anti-Hyperglycemic Activities

Diabetes is a chronic disease responsible for kidney failure, blindness, amputations, stroke, and increased risk of coronary artery disease. Orange-footed sea cucumber (*C. frondosa*) extracts have demonstrated potential anti-diabetic and glucose-lowering activity. Hu et al. [104] isolated EPA enriched phosphatidylcholine from *C. frondosa* and suggested notable anti-hyperglycemic properties through up-regulating phosphatidylinositol 3 kinase (PI3K)/protein kinase B (PKB) signal pathway mediated by insulin. Moreover, phosphatidylcholine remarkably reduced blood glucose level, while the opposite scenario was observed in glycogen synthesis and insulin secretion in diabetic rats. In addition, RT-PCR analysis showed that phosphatidylcholine significantly stimulated the expressions of glycometabolism-related genes of the insulin receptor, protein kinase B, phosphoinositide 3-kinase, insulin receptor substrate-1, and glucose transporter 4 in gastrocnemius. Another study revealed the isolation of fucoidan from *C. frondosa*, and examined its anti-hyperglycemic activities via activating phosphatidylinositol 3 kinase (PI3K)/protein kinase B (PKB) pathway and glucose transporter 4 (GLUT4) in skeletal muscle and adipose tissue [70]. Results indicated that fucoidan notably decreased fasting blood glucose and insulin levels, while the opposite was true in insulin and glucose tolerance in insulin-resistant mice. Furthermore, RT-PCR analysis showed that fucoidan raised the insulin receptor substrate 1, mRNA expressions of insulin receptors, PI3K, PKB, and GLUT4. 

Hu et al. [122] isolated fucosylated chondroitin sulfate from the body wall of *C. frondosa*, and investigated the effects of a combination of FCS and rosiglitazone (RSG) on glucose metabolism in high-fat high-sucrose fed mice. Results suggested that the combination of FCS and RSG significantly improved liver weight, body weight gain, fasting blood glucose levels, serum insulin levels, glucose tolerance on an oral glucose tolerance test, hepatic glycogen content, and homeostasis model assessments indices compared to FCS or RSG alone. Hu et al. [123] also determined the effects of fucosylated chondroitin sulfate (FCS) isolated from *C. frondosa* on inhibition of pancreatic islets apoptosis in insulin-resistant mice and suggested that FCS notably repaired high-fat high-sucrose diet injured pancreatic islets, reduced insulin, blood glucose, TNF-α levels, and raised adiponectin level. Similarly, Hu et al. [58] examined the effects of a fucoidan from *C. frondosa* on pancreatic islets and reported that it significantly prevented high-fat high-sucrose diet (HFSD)-injured pancreatic islets, decreased insulin, blood glucose, and TNF-α levels, while adiponectin level was increased. 

### 6.5. Immunomodulatory Activities

Frondoside A (monosulfated pentaoside) is a glycoside compound identified from *C. frondosa* with immunomodulatory activities when administered in subtoxic doses [100]. Moreover, the lysosomal activity of macrophages in treated animals increased significantly compared to vehicle-treated controls when frondoside A (0.2-μg) was administered intraperitoneally to Balb C mice. Therefore, frondoside A raised the cell-based immunity, which is responsible for increasing the innate immune responses and inhibiting tumor growth. Another study by Kale et al. [56] revealed the immunomodulatory effects of sea cucumber-derived (*C. frondosa*) sulfated polysaccharide extracts and reported that the monosaccharide composition included *N*-acetylneuraminic, *N*-acetylglucosamine, mannose, galactose, fucose, and *N*-acetylgalactosamine (40:13:12:12:9:8:3), which were found to be active in maturing dendritic cells and leading to increased Th17 signaling. On the other hand, Janakiram et al. [118] suggested that frondanol A5 raised GILT expression as well as macrophage phagocytosis and declined inflammatory angiogenic molecules, which may increase the innate immune systems of the treated mice. 

### 6.6. Anti-Inflammatory Activities

Anti-inflammatory agents are substances that reduce inflammation. Sea cucumber exhibits potential anti-inflammatory properties due to the presence of biologically active compounds, including bioactive peptides [5]. In addition, sulfated polysaccharides (*N*-acetylneuraminic acid) from *C. frondosa* may influence dendritic cell maturation, thus ultimately showing anti-inflammatory activities [56]. In another study, Stefaniak-Vidarsson et al. [94] stated that sulfated polysaccharides (FCF-1, FCF-2, and FCF-3) from *C. frondosa* might have functional activities toward THP-1 macrophages and anti-inflammatory properties. Moreover, ORAC was evaluated, and cell culture media was collected and examined for the presence of anti-inflammatory (IL-10) and pro-inflammatory (TNF-α and IL-6) cytokines. Additionally, FCF-1, FCF-2, and FCF-3 (0.1–100 µg/mL) induced levels of TNF-α, IL-10, and IL-6 proofing their functional activity towards THP-1 macrophages. Besides, Subramanya et al. [124] reported that frondanol, a non-polar extract obtained from *C. frondosa*, demonstrated anti-inflammatory activity in a DSS (dextran sodium sulfate)-induced mouse model of colitis. 

### 6.7. Antioxidant Activities

Nowadays, marine-based natural antioxidants have gained much attention. In particular, sea cucumber is considered a possible source of valuable antioxidants with multiple biological activities. Antioxidant properties can be analyzed by a variety of assays with various mechanisms of action, mainly hydrogen atom transfer (HAT), reducing power, single electron transfer (SET), and metal chelation [125]. Hydroxyl radical can react with proteins, amino acids, and DNA, thus triggering lipid peroxidation [126]. Consequently, the elimination of the hydroxyl radical is possibly one of the most effective defenses of a system against oxidation [127]. Besides, DPPH is a stable radical and responsible for scavenging free radicals through hydrogen or electron-donating mechanisms to become a stable diamagnetic molecule [127]. Yan et al. [95] analyzed the antioxidant activity of *C. frondosa* by using different enzymes and stated that the highest amount of hydroxyl radical scavenging activity (27.4%) and DPPH radical scavenging activity (14.42%) were obtained by trypsin and Flavourzyme followed by Alcalase. In addition, Mamelona et al. [93] prepared protein hydrolysates from echinoderm byproducts and reported that the viscera of *C. frondosa* how the highest ORAC value (421 µmol of Trolox equivalents/ g) compared to the digestive tract of green sea urchin. Moreover, hydrolysates exhibited the inhibition of lipid oxidation (> 50%), and the reason for antioxidant activity could be due to the presence of antioxidant peptides in the hydrolysates. In another study, Wu et al. [128] examined the effect of eicosapentaenoic acid-enriched phospholipids (EPA-PL) from *C. frondosa* on oxidative injury in PC12 cells induced by *tert*-butyl hydroperoxide and hydrogen peroxide. Results demonstrated that EPA-PL decreased the leakage of lactate dehydrogenase and raised the intracellular total antioxidant activity and superoxide dismutase capacity compared with *tert*-butyl hydroperoxide or hydrogen peroxide. In addition, it revealed the neuroprotective activities by virtue of its antioxidant property, which could be accomplished by inhibiting the mitochondria-dependent apoptotic pathway. 

### 6.8. Antimicrobial Activities

The extracts of orange-footed sea cucumber have shown potential antimicrobial activities due to the presence of several crucial enzymes. Haug et al. [129] examined the antimicrobial activities of different body parts of orange-footed sea cucumber and reported that the eggs of *C. frondosa* showed significantly higher antimicrobial properties compared to the body wall extracts. Additionally, hemolytic activity was observed in the body wall extracts of sea cucumber. Antimicrobial activity was also noticed in the coelomocytes, mainly against the Gram-positive bacteria, suggesting that marine echinoderms, particularly *C. frondosa* are a possible source for the discovery of novel antibiotics. Similarly, Beauregard et al. [32] identified a novel antimicrobial peptide from the coelomic fluid of *C. frondosa* and found it to have a broad range of antimicrobial activity against both Gram-positive and Gram-negative bacteria. In another study, Tripoteau et al. [130] evaluated the antiviral activities of enzymatic hydrolysates extracted from the byproducts of *C. frondosa*, and claimed that it could serve as a potential source of an antiviral agent against *Herpes Simplex* virus 1. Sea cucumber triterpene glycosides do not show antibacterial activity due to their targets are 5,6-unsaturated cell membrane sterols. However, most sea cucumber glycosides are powerful antifungal agents. Unfortunately, frondoside A was never tested on the antifungal activity even though its structure is very close to potent antifungal components [131]. 

### 6.9. Anti-Angiogenic Activities

Anti-angiogenic agents can destroy the network of blood vessels needed by metastasizing tumors. Sea cucumber is a possible source of anti-angiogenic compounds, particularly *C. frondosa*. Fucosylated chondroitin sulfate (FCS) is one of the most important constituents with anti-angiogenic activity. Collin [132] isolated FCS from flower and body wall of *C. frondosa* and reported FCS has a relatively higher anti-angiogenic activity compared to hydrocortisone/ heparin as a positive control and even shark cartilage CS6. In another study, Attoub et al. [115] described that frondoside A is responsible for angiogenesis as indicated by reduced CD31 staining in LNM35 lung cancer xenografts without toxic side effects. 

### 6.10. Antihypertensive Activities

Much attention has recently been paid to the potential antihypertensive and angiotensin converting enzymes (ACE) inhibitory activities of sea cucumber. The body wall protein of sea cucumber shows antihypertensive properties. Hamaguchiet al. [133] evaluated the antioxidant and antihypertensive properties of differently processed Icelandic sea cucumber. *C. frondosa* was separated as skin, muscle, respiratory tract, and digestive tract, and processed in several ways for aqueous extraction and hydrolysis to evaluate the ACE inhibitory activity, ORAC value, metal ion chelating activity, and reducing power. Results demonstrated that aqueous extract had significantly higher ACE inhibition compared to the hydrolysates. Moreover, hydrolysates exhibited higher ORAC values than the aqueous extracts. 

### 6.11. Other Activities

Orange-footed sea cucumber demonstrates potential biological activities, in addition to those described already. For example, Zhang et al. [134] claimed that the EPA enriched phospholipids of *C. frondosa* are responsible for fat burning and could be used as functional food for treating obesity disorders. In another study, Hui et al. [135] described that sphingolipid from *C. frondosa* was able to inhibit proliferation and differentiation of 3T3-L1 cells (derived from mouse 3T3 cells) by long-chain bases (LCB) compared to cerebroside and ceramide. Similarly, Jia et al. [136] isolated glucocerebroside series (CFC-1, CFC-2, and CFC-3), ceramides, and long-chain bases from *C. frondosa*, and reported that glucocerebroside series, ceramides, and long-chain bases render an inhibitory effect on cell proliferation. Additionally, CFC-3 was the most effective compound compared to other glucocerebrosides for HepG-2 cell viability. In another experiment, Tian et al. [137] examined the anti-adipogenic activities of long-chain bases from *C. frondosa*, and showed suppressed adipocyte differentiation and expressions of CCAAT/enhancer binding proteins as well as peroxisome proliferators-activated receptor γ. On the other hand, Yan et al. [95] produced various protein hydrolysates from *C. frondosa* viscera by using several commercial enzymes (trypsin, Alcalase, Flavourzyme, Neutrase, papain, and bromelain), and reported that the hydrolysates prepared by using Flavourzyme and Neutrase had better foaming properties (foaming capacity and foaming stability). In addition, papain and bromelain derived hydrolysates demonstrated better emulsifying activity than those of other proteases. Besides, Lin et al. [138] prepared *C. frondosa* hydrolysate by enzymatic hydrolysis and analyzed the anti-aging effects on fruit flies and D-galactose-induced aging mice. The result suggested that the *C. frondosa* hydrolysate contained low molecular weight peptides which prolonged the lifespan of fruit flies and aging mice via Klotho expression, prevention of lipid peroxidation and protein oxidation, increasing the activities of superoxide dismutase (SOD) and glutathione peroxidase (GSH-Px), and down-regulating the acetylcholinesterase activity.

## 7. Prospects, Challenges, and Future Aspects of Atlantic Sea Cucumber

Many research efforts have been underway on sea cucumber due to its potential use in pharmaceutical, functional food, nutraceutical, cosmeceutical, and agrochemical industries. However, *C. frondosa* is still a relatively underexplored species and its bioactive compounds and their functional properties are yet to be characterized. According to DFO [45], growth-sex, recruitment processes, age at maturity, natural mortality, harvesting method, ecosystem structure and function, population structure, and sustainable exploitation rates of *C. frondosa* are still unknown and need to be established. Since sea cucumber contains 70%–90% water, it can autolyze after leaving seawater, which makes its preservation and transportation very difficult [139]. In order to maintain better nutritional and organoleptic properties, special care should be exercised for the storage and transport of fresh animals after harvesting. Moreover, the relation among age, sex, and harvesting time in terms of bioactive compounds is still ambiguous and needs to be identified. Furthermore, body walls of sea cucumber are the primary sources of bioactive compounds, but it is extremely challenging to separate the muscle, inner dermis, or meat. Therefore, using chemicals and enzymes or high pressure can serve as effective means to dissect this animal properly. On the other hand, the body wall, which accounts for up to 50% of the total body weight, is immediately stored in seawater prior to further processing and sold as market products. However, the internal organs, including gonads, intestines, and respiratory trees are usually discarded as waste, and not much have been done for their potential use. Consequently, future research should be directed towards utilization of sea cucumber discards for the extraction of potential value-added products and their application in nutraceuticals, pharmaceuticals, and functional foods. For example, during processing of sea cucumber, blanched water is currently disposed; however, it could be dehydrated to a powder or concentrated and applied in fortified foods such as canned soup or functional bread. In addition, it can be used as a food ingredient or supplement in cereal-based products and snack foods, or as flavor binding and gelatin products. 

On the other hand, East Asians use sea cucumber as a traditional folk medicine, however, claims need to be supported by scientific findings and pre-clinical and clinical research in *C. frondosa*. Although a few medicinal properties of sea cucumber have been established but there is a need to modify the methods employed to validate them in vivo. In addition, several in vitro reports have indicated that *C. frondosa* has potential anticoagulant, anti-angiogenic, anticancer, anti-inflammatory, antihyperglycemic, ACE inhibitory, antitumor, and other activities [5]. So far, numerous compounds have been isolated from sea cucumber, but there is a need to fully identify, characterize, and purify them; these include phenolics, alkaloids, peptides, and other valuable components. Another possible exploitation of *C. frondosa* is to develop functional foods, such as components that are required for food fortification or substitution. However, functional foods represent one of the main challenges, not only for pharmacological efficacy as medicine but also sensory quality of the products must be fully considered. Additionally, the development of sea cucumber protein hydrolysates and further investigation into collagen remains needs to be explored. 

Due to the antimicrobial properties of *C. frondosa*, sea cucumber extract/powder could be applied to beverages and baked products (e.g., bread and cookies) as a natural antimicrobial preservative for shelf life extension. Sea cucumber body wall is the major source of collagen due to its lower level of lipid and a higher level of protein compared to most other foods [23]. However, claims need to be proven in *C. frondosa* and more research is necessary to isolate, identify, and quantify the individual peptides and their amino acid sequences. Moreover, research into the extraction and further evaluation of collagen from sea cucumber must also be perused in order to examine their potential utilization in nutraceutical applications. Therefore, sea cucumber-derived bioactive peptides could offer a safe and reliable source of ingredients for human health promotion and disease risk reduction. Additionally, due to anti-aging, skin whitening, antimicrobial, and wound healing activities of sea cucumber [38], further studies are needed to isolate and identify the specific compounds for potential use in cosmeceutical applications. Stonik et al. [140], however, reviewed the toxins of sea cucumbers (holothuroids) and reported that they are mostly related to triterpene oligoglycosides with one or more sulfate groups in the carbohydrate chains, but the adverse effects of *C. frondosa* on human health or environment are not documented as of yet.

## 8. Conclusions

Sea cucumber (*C. frondosa*) is an excellent source of bioactive compounds such as chondroitin sulfate, saponins, amino acids, glycosides, collagen, peptides, phenolics, and gelatin. However, understanding its detailed chemical structure, isolation and identification, and bioactivities is still not fully explored, and remains to be established using different models, including clinical methods. There is a great potential for enhancing the economic value of *C. frondosa* by full exploitation and application in functional food, pharmaceutical, nutraceutical, and cosmeceutical industries. Therefore, the broad range of bioactive compounds and their possible extraction, purification, and identification methods as well as potential applications in the field of health care, cosmetics, pharmaceuticals, and nutraceuticals presented in this overview may help researchers to fully utilize this valuable resource in a sustainable manner.

## Figures and Tables

**Table 1:** Current sea cucumber (*Cucumaria frondosa*) products in the markets.

Product Name	Brand/Manufacturer	Price (US$)
PEACE PAVILION dried sea cucumber	Peace Pavilion	$150.00/LB
Canadian wild freshly dried sea cucumber	NorthBay Foods	$135.00/LB
Dried Canada wild sea cucumber	Arctica Food	$98.00/LB
Dried sea cucumber- butterfly cut/whole cut	Atlantic Sea Cucumber	$55.00–75.00/LB
Arctic dried sea cucumber	Naturally North	$65.00/LB
Sea cucumber liquid extract	Hawaii Pharm	$20.00/oz.
Dried *Cucumaria frondosa* flower	NorthBay Foods	$15.00/LB
Canadian sea cucumber (whole/split east coast)	SEACOO	N/A

Note: The information was obtained from www.amazon.com and manufacturer website, the search keywords are “*Cucumaria frondosa*” and “*Cucumaria frondosa* product”.

**Table 2:** Total amino acid profile and fatty acid composition of fresh Atlantic sea cucumber.

Amino acids	Sea cucumber with viscera (mg/g) ^a^	Body wall (mg/g) ^a^	Viscera (%) ^b^	Fatty Acids	Sea cucumber with viscera (%) ^a^	Body wall (%) ^a^	Viscera (%) ^b^
Valine	16.8	19.7	5.4	14:00	1.8	1.88	10.1
Methionine	9.4	10.3	2.3	15:00	4.03	2.18	0.3
Isoleucine	12.6	13.9	4.7	16:00	2.83	2.33	13.3
Leucine	28.3	30.8	7.2	16:1 n-7	7.36	5.75	6
Phenylalanine	13.1	17.8	3.5	17:1 n-7	2.44	3.87	N/A
Histidine	3.3	2.8	2.3	18:00	4.2	5.41	2.1
Threonine	10.9	12.9	5	18:1 n-9	3.72	2.43	4.9
Lysine	30.6	29.1	6.6	18:1 n-7	3.37	3.52	N/A
Aspartic acid	27.8	39.1	10	20:1 n-9	1.66	4	N/A
Glutamic acid	57.5	66.4	14.3	20:3 n-3	2.54	5	2.5
Serine	15.5	19.3	4.3	20:5 n-3	43.2	46.1	17.1
Glycine	29.8	56.1	8	22:00	2.09	1.95	0
Alanine	23.2	32	6.6	22:1 n-9	3.34	2.25	2.5
Arginine	24.7	30.1	9.1	22:6 n-3	5.81	4.96	0.3
Proline	17.3	24	4	20:2 n-6	N/A	N/A	2.2
Tyrosine	13.4	15.6	5	20:4 n-6	N/A	N/A	10.4

^a^ Zhong, Khan, and Shahidi [54]; ^b^ Mamelona, Saint-Louis, and Pelletier [55].

**Table 3:** Biologically active compounds of Atlantic sea cucumber and their functions.

Bioactives	Body Parts	Biological and Medicinal Effects	Extraction and Isolation Method	References
Fucosylated chondroitin sulfate	Body wall	Antithrombotic, anticoagulant, anticancer, anti-inflammatory, antitumor, antidiabetic, anti-osteoarthritis, alleviates inflammation, alleviates pain, and improve immune system	Enzymatic (papain/ Alcalase) hydrolysis followed by precipitation (cetylpyridinium chloride/ ethanol/ sodium hydroxide/ tricholoracetic acid)	[57,93,99]
Collagen	Body wall	Antihypertension, antiaging, anti-wrinkle, alleviates skin problems, and wound healing	A divalent cation chelator (EDTA) followed by extraction in water	[79,80]
glycosides (saponins)	Body wall	Antibacterial, antifungal, antiviral, antitumor, anticancer, antiangiogenic, and photo-protective	Isopropyl alcohol/ water extraction and refluxing with chloroform/ methanol/ethanol	[83,100,101]
Fucoidan	Body wall	Anticoagulant, antibacterial, antiaging, anti-hyperglycemic, lowering blood glucose level, and photo-protective	Hydrolysis with papain and precipitation with cetylpyridinium chloride	[58,70]
Phenolic compounds	Body wall, tentacles, and viscera	Antioxidants and antibacterial	Solvent extraction (methanol), water, organic solvent (ethyl acetate) and a mixture of water/ miscible organic solvent (acetonitrile)	[16,54]
Cerebrosides	Body wall	Anticancer, anti-inflammatory, and anti-adipogenic activity	Solvent extraction (65% ethanol) and isolated by High-performance liquid chromatography (HPLC), extracted by chloroform/ methanol using high speed counter-current chromatography	[19,102,103]
Amino acid	Body wall, tentacles, and viscera	Anti-fatigue, repairing tissue, nutritional storage, and wound healing	Reversed phase HPLC	[54,55]
Protein (bioactive peptide)	Body wall	Antimicrobial	Fractionated utilizing ammonium sulfate precipitation and analyzed by size exclusion chromatography	[32]
Vitamin and minerals	Body wall, tentacles, and viscera	Cosmeceutical properties, promote healthy growth and metabolism, lower the blood sugar level	Association of Official Analytical Chemists (AOAC)-and inductively coupled plasma mass spectrometry (ICP-MS)	[55,93]
Omega-3 (EPA)	Body wall, tentacles, and viscera	Anti-hyperglycemic, decrease cholesterol, and protect the heart	Solvent extraction (methanol: chloroform: water) and analyzed by gas chromatography (GC)/ HPLC	[50,54,55,104]

**Table 4:** Effects of triterpene glycosides on various cancer cells and tumors.

Compounds	Activities	Type of Cancer	References
Frondoside A	Anti-proliferation, antitumor, anti-anti-metastatic, apoptosis, p21 increase, increased caspase 3and 7, migration and invasion, increase in p53, caspase 3/7, decrease ERK ½, decrease cell viability, cell cycle arrest, inhibit colony formation, angiogenesis, and inhibit pro-survival autophagy	Pancreatic cancer cells, xenografts, lung and breast cancer, lung cancer xenografts, breast cancer, breast cancer xenografts, non-small lung carcinoma, leukemia, cervical cancer, and urothelial carcinoma (HT-1197, RT112, and RT4)	[56,101,108,109,110,111,112,113,114,115,116,117]
Frondanol A5	Anti-proliferation, inhibition of cell cycle, induce apoptosis, aberrant crypt inhibition, p21 increase, DNA fragmentation, apoptosis, G2/M inhibition, inhibition of small intestinal and colon tumors, increase in GILT expression, macrophage phagocytosis, decrease expression of cyclin A, cyclin B, and cdc25c	Pancreatic cancer cells, AOM-induced rat colon cancer model, human colon cancer cells HCT116, Apc^Min/+^ colon cancer model, colon cancer, and pancreatic cancer (AsPC-1 and S2013)	[110,111,118]
Frondoside A and B	Induce apoptosis, inhibition of cell cycle, antitumor, anti-anti-metastatic, decrease cell viability, cell cycle arrest, and inhibit colony formation	Pancreatic cancer (AsPC-1 and S2013), non-small lung carcinoma, hepatocellular liver carcinoma, leukemia, breast adenocarcinoma, Luc-2 breast cancer, and breast cancer	[101,108,109,112,115,116]
Frondoside A + gemcitabine	Tumor inhibition, apoptosis, necrosis, increased caspase 3,7 and 9	Pancreatic cancer xenografts	[115]
Frondoside A + cisplatin	Antitumor	Lung cancer xenografts	[113]
Frondoside A + paclitaxel	Cytotoxic	Breast cancer cells	[111]
